# Multilevel data integration and molecular docking approach to systematically elucidate the underlying pharmacological mechanisms of Er-Zhi-Wan against hepatocellular carcinoma

**DOI:** 10.18632/aging.204369

**Published:** 2022-11-07

**Authors:** Shaoyan Zheng, Botao Pan

**Affiliations:** 1 Affiliated Foshan Maternity and Child Healthcare Hospital, Southern Medical University, Foshan 528000, P.R. China; 2 Traditional Chinese Medicine Department, Affiliated Foshan Maternity and Child Healthcare Hospital, Southern Medical University, Foshan 528000, P.R. China

**Keywords:** Er-Zhi-Wan (EZW), hepatocellular carcinoma (HCC), cell cycle, cellular senescence, molecular docking

## Abstract

As a multicomponent, multitarget empirical therapy, traditional Chinese medicine (TCM) has been used clinically in Asia for thousands of years. Due to this unique feature, TCM therapy is considered a promising therapeutic strategy for the treatment of hepatocellular carcinoma (HCC). Er-Zhi-Wan (EZW), a well-known TCM formula containing two herbs, *Fructus Ligustri Lucidi* (FLL, Nü-Zhen-Zi) and *Ecliptae Herba* (EH, Mo-Han-Lian), is commonly used in clinical practice to prevent and treat liver diseases. Modern pharmacological studies have shown that both EH and FLL can inhibit HCC proliferation. However, the pharmacological mechanism, potential targets, and clinical value of EZW in inhibiting HCC have not been fully elucidated. We used multilevel databases (Gene Expression Omnibus (GEO), Traditional Chinese Medicine Systems Pharmacology (TCMSP), High-throughput Experiment- and Reference-guided database (HERB), and SwissTargetPrediction) to show that EZW suppresses HCC through 19 active components acting on 66 potential targets. Enrichment analysis revealed that EZW mainly regulates HCC progression through various metabolic pathways, the cell cycle, and cellular senescence. Furthermore, we used The Cancer Genome Atlas (TCGA)-LIHC database to analyze the expression patterns and clinical characteristics of cellular senescence-related genes and identified CDK1, CDK4, CHEK1, and G6PD as key therapeutic molecular targets in EZW-suppressed HCC. Molecular docking revealed that EZW could exert its anti-HCC effect by binding various active components to the above cellular senescence-related genes and regulating their activities. In conclusion, we systematically revealed the potential pharmacological mechanisms and molecular targets of EZW against HCC based on multilevel data integration and a molecular docking strategy.

## INTRODUCTION

Worldwide, liver cancer is one of the most common cancers, and its morbidity and mortality are on the rise [1, 2]. It has a mortality-to-morbidity ratio of 0.91 and is 2.3 times more common in men than in women [3]. Notably, the outlook for patients is even grimmer in Asia, where 72% of new cases are reported to be diagnosed (over 50% in China), with five-year survival rates as low as 12% [4, 5]. Hepatocellular carcinoma (HCC) is the most common type of primary liver cancer and often develops from chronic liver disease caused by hepatitis B virus or hepatitis C virus infection, alcoholism, or metabolic syndrome [6]. Currently, surgical resection, liver transplantation, and locoregional therapy (including radiofrequency ablation) are recommended as curative treatments for only one-third of HCC patients. The remaining 60%–70% of patients receive noncurative treatments, such as molecularly targeted agents, monoclonal antibodies, or immune checkpoint inhibitors, as initial therapy [7]. However, due to the heterogeneity and complexity of HCC, most patients are diagnosed at an advanced stage; therefore, systemic therapy is often recommended as the standard of care [8]. Although it is more effective than monotherapy, it is only suitable for use in a small number of patients and is associated with severe toxicity [9].

The pathological mechanism of HCC is very complex, and multiple targets and signaling pathways are involved in its development [10]. This complexity requires the development of a therapeutic strategy that modulates multiple targets in HCC to improve patient outcomes. As a multicomponent and multitarget empirical therapy, traditional Chinese medicine (TCM) has been recognized worldwide in recent years for its multitarget synergistic intervention effect on HCC. Clinically, as an adjuvant drug, TCM has shown good efficacy in HCC patients, significantly reducing the incidence, preventing the recurrence, and improving the overall survival of HCC patients [11–14]. In addition, modern pharmacological studies have shown that TCM or TCM-derived natural medicines can effectively suppress the development and progression of HCC *in vitro* and *in vivo* [15–18]. Due to this unique feature, TCM is considered a promising therapeutic strategy for the treatment of complex diseases, including liver cancer.

Er-Zhi-Wan (EZW), a well-known TCM formula, was first recorded in the ‘Fu Shou Jing Fang’ in the Ming Dynasty [19]. It consists of an equal weight mixture of two herbs, *Fructus Ligustri Lucidi* (FLL, Nü-Zhen-Zi) and *Ecliptae Herba* (EH, Mo-Han-Lian). According to Chinese medicinal theory, EZW is commonly used clinically in China for the treatment of liver-kidney yin deficiency syndrome (LKYDS), which is a pathological and diagnostic pattern caused by an imbalance of yin and yang and is more common in primary liver cancer, diabetes, and hypertension [20]. Therefore, EZW is commonly used clinically in China to prevent or treat various liver and kidney diseases. Yao et al. [21] uncovered the hepatoprotective effect of EZW based on a metabolomic strategy. Hu et al. [22] revealed that FLL extract induced apoptosis and cellular senescence in human hepatoma cells by upregulating p21, confirming that FLL is a potential anticancer herb for the treatment of HCC. Moreover, our previous study showed that EH extract could inhibit the proliferation of HCC cells by inhibiting PI3K-AKT signaling [18]. However, few studies have comprehensively investigated the molecular mechanisms involved in EZW in the treatment of HCC.

**Figure 1:**
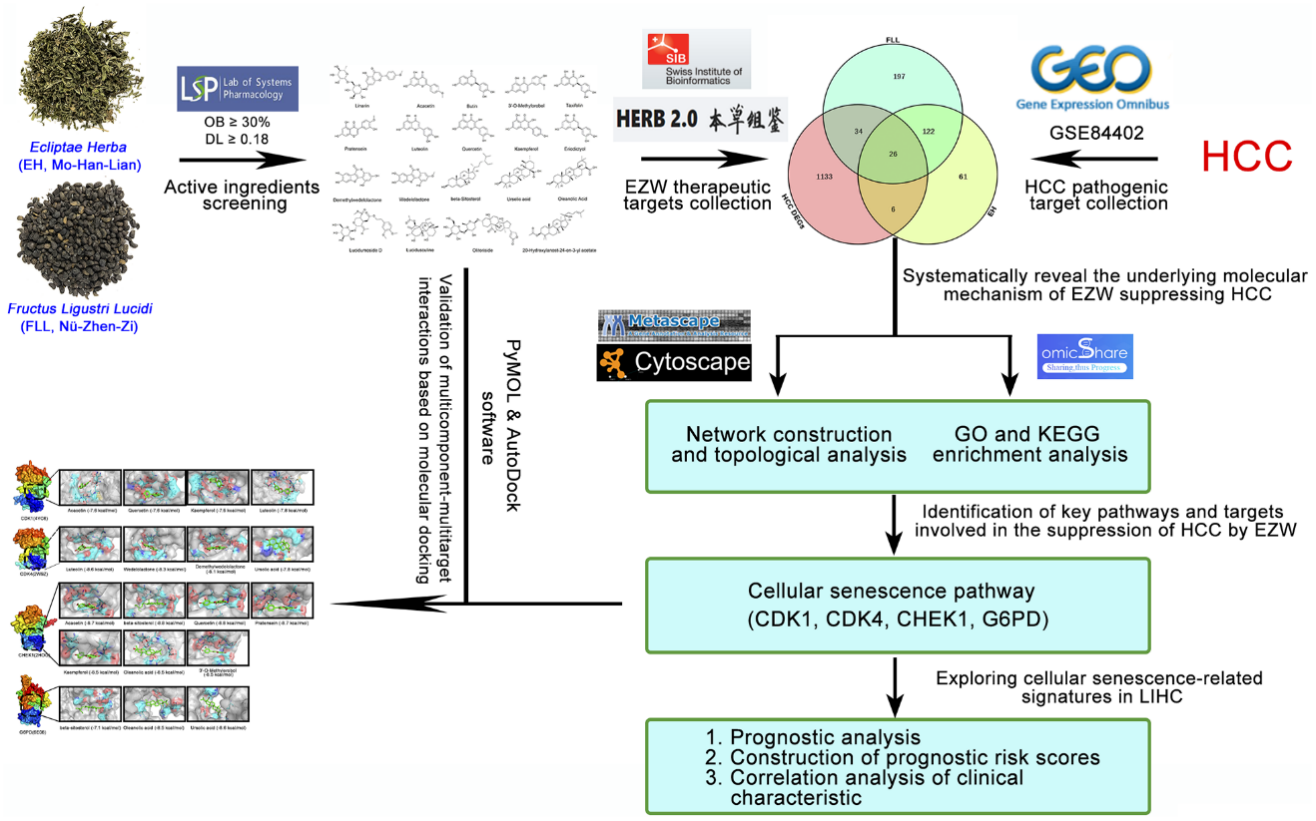
Flowchart of the analytical procedures of the study.

Since TCM prescriptions comprise many kinds of herbs and contain many kinds of ingredients, it is difficult to systematically and comprehensively study the pharmacological mechanism of TCM with the existing experimental methods. With the successful establishment of multiple biological databases and the rapid development of systems biology, the emergence of network pharmacology has brought great opportunities for breakthroughs in TCM research [23, 24]. To date, this method has been successfully used to elucidate the multitarget efficacy of TCM in the treatment of various diseases, effectively bridging the gap between Western medicine and Chinese medicine [25, 26]. In this study, we employed various biological databases and biocomputational approaches to investigate the pharmacological network involved in EZW in the treatment of HCC to predict potential molecular targets and pharmacological mechanisms. The overall research flowchart is shown in Figure 1.

## RESULTS

### Identification of pathological genes in HCC

**Figure 2:**
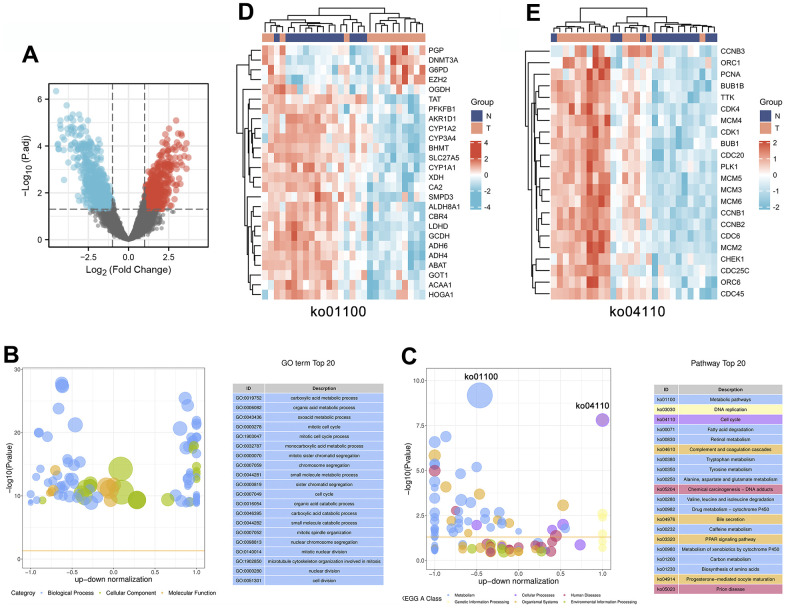
**Identification and enrichment analysis of differentially expressed genes in 14 pairs of HCC tissues and corresponding noncancerous tissues.** (**A**) The expression patterns of the DEGs are shown in volcano plots. Red and blue points represent upregulated genes (log2FC ≥ 1) and downregulated genes (log2FC ≤ -1), respectively, while gray represents genes with no significant difference in expression (P.adj < 0.05). (**B**, **C**) Bubble plot showing the top 20 GO (**B**) and KEGG (**C**) enrichment analysis results. The larger the ordinate value in the bubble chart, the more significant the corresponding GO or KEGG result is. The abscissa represents the normalized upregulation and downregulation value (the ratio of the difference between the number of upregulated genes and the number of downregulated genes to the total number of differentially expressed genes). The higher the value is, the higher the number of upregulated genes enriched in the GO/KEGG pathway results; conversely, the lower the value is, the higher the number of downregulated genes enriched in the GO/KEGG pathway results. (**D**, **E**) Heatmaps showing the expression patterns of genes involved in the cell cycle (ko04110) or metabolic pathways (ko01100).

To identify which genes are involved in the progression of HCC, we analyzed the Gene Expression Omnibus (GEO) dataset GSE84402 to identify genes that were differentially expressed in 14 pairs of HCC tissues and corresponding noncancerous tissues. As shown in the volcano plot, a total of 1199 differentially expressed genes (DEGs) were identified in these 14 pairs of liver tissues, of which 632 genes were upregulated and 567 genes were downregulated in cancerous tissues compared with noncancerous liver tissues (Figure 2A and Supplementary File 1). It is speculated that the progression of HCC involves extensive and complex pathological gene regulation.

### Gene Ontology (GO) and Kyoto Encyclopedia of Genes and Genomes (KEGG) enrichment analyses of DEGs in HCC

To further investigate the underlying molecular mechanisms involved in HCC progression, we performed enrichment analysis on 1199 DEGs, including GO and KEGG analyses. We used bubble plots to display the top 20 GO (Figure 2B) and KEGG (Figure 2C) enrichment analysis results. GO functions can be divided into three categories: biological process (BP), molecular function (MF), and cellular component (CC), and the top 20 GO annotation results show that these DEGs are mainly enriched in the BP category. These BPs mainly involve metabolic and cellular processes, including the following: “carboxylic acid metabolic process (GO:0019752)”, “organic acid metabolic process (GO:0006082)”, “oxoacid metabolic process (GO:0043436)”, “mitotic cell cycle (GO:0000278)”, “mitotic cell cycle process (GO:1903047)”, “cell cycle (GO:0007049)”, and “cell division (GO:0051301)”.

In addition, the results of the top 20 KEGG pathways indicated that the progression of HCC is closely related to 6 categories, including metabolism, cellular processes, human diseases, genetic information processing, organismal systems, and environmental information processing. In particular, 12 of the 20 pathways were involved in the metabolism category, including “Metabolic pathways (ko01100)”, “Fatty acid degradation (ko00071)”, “Retinol metabolism (ko00830)”, “Tryptophan metabolism (ko00380)”, “Tyrosine metabolism (ko00350)”, “Alanine, aspartate and glutamate metabolism (ko00250)", "Valine, leucine and isoleucine degradation (ko00280)”, “Drug metabolism – cytochrome P450 (ko00982)”, “Caffeine metabolism (ko00232)”, “Metabolism of xenobiotics by cytochrome P450 (ko00980)”, “Carbon metabolism (ko01200)”, and “Biosynthesis of amino acids (ko01230)”. Notably, metabolic pathways (ko01100) were enriched with multiple downregulated DEGs, including multiple metabolic enzymes (PFKFB1, AKR1D1, CYP1A2, XDH, ALDH8A1, LDHD, GCDH, ADH6, ADH4, ABAT, and HOGA1). The heatmap results showed that abnormal changes in various metabolic enzymes occurred during the progression of HCC, which suggested that abnormal expression of metabolic enzymes affected the development of HCC (Figure 2D). Furthermore, HCC progression is regulated by the cell cycle (ko04110), and multiple upregulated DEGs were found to be significantly enriched in this pathway, including multiple cell cycle-related regulatory genes (CCNB3, PCNA, CDK1, CDK4, CCNB1, CCNB2, and CHEK1). The combined heatmap analysis indicated that the cell cycle is abnormally activated in HCC, leading to the indefinite growth of HCC (Figure 2E).

### Screening of active compounds and potential therapeutic targets of EZW

**Figure 3:**
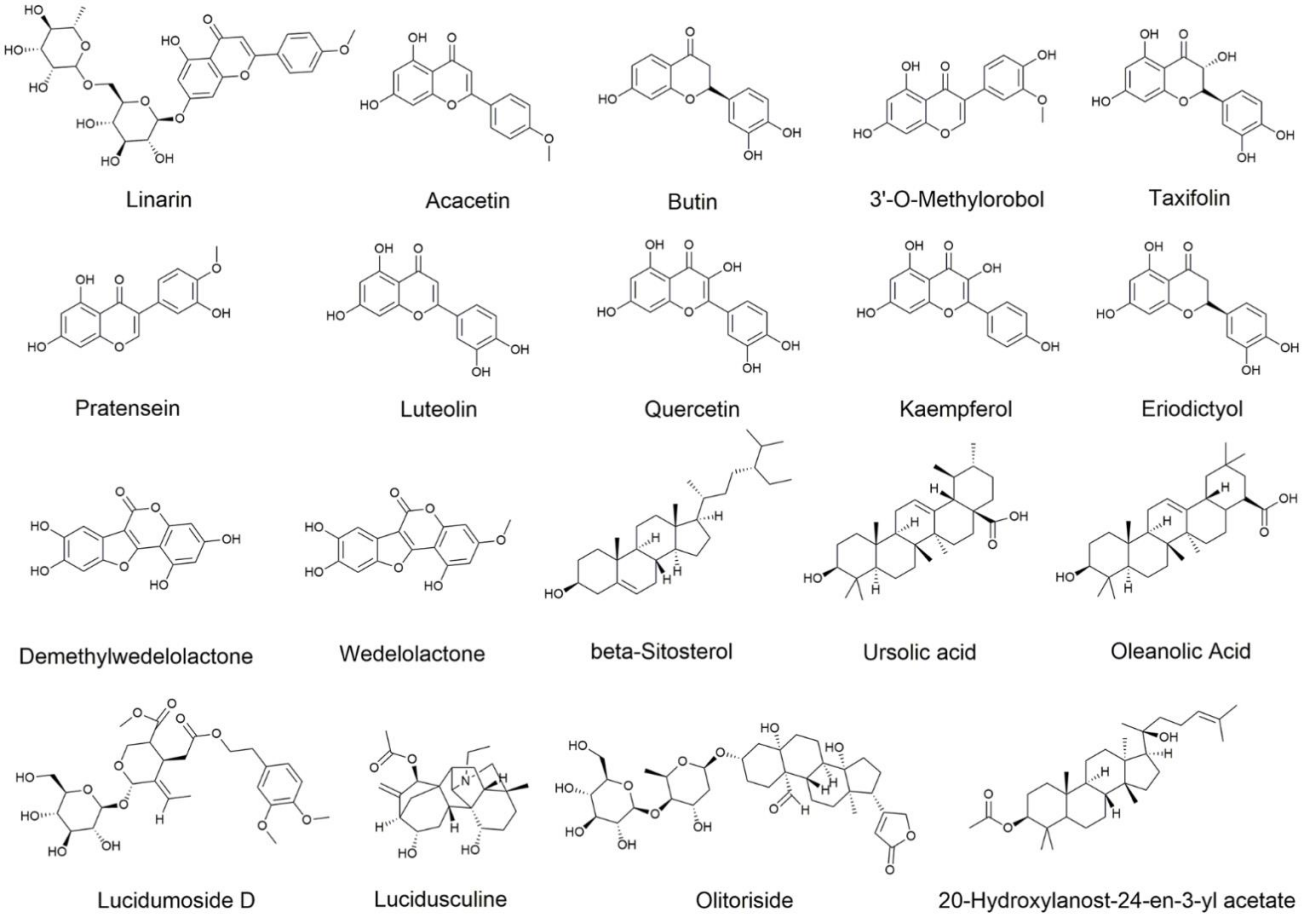
Chemical structures of 19 active ingredients of EZW.

EZW is produced by mixing *Fructus Ligustri Lucidi* and *Ecliptae Herba* in equal proportions. According to the two criteria of drug-likeness (DL) ≥ 0.18 and oral bioavailability (OB) ≥ 30% [27, 28], a total of 9 active ingredients in EH and 10 active ingredients in FLL were identified in TCMSP. Among them, studies have shown that both oleanolic acid and ursolic acid are active. components of FLL, but they were not included in the TCMSP screening analysis because they did not meet the DL and OB conditions [29]. Therefore, to increase the credibility of the study, these two active ingredients were included in the list of active ingredients of FLL By combining the active ingredients of EH and FLL, it was revealed that EZW comprised a total of 19 active ingredients, of which the two herbs each contained quercetin and luteolin (Supplementary File 2). The chemical structures of these active compounds are shown in Figure 3.

**Figure 4:**
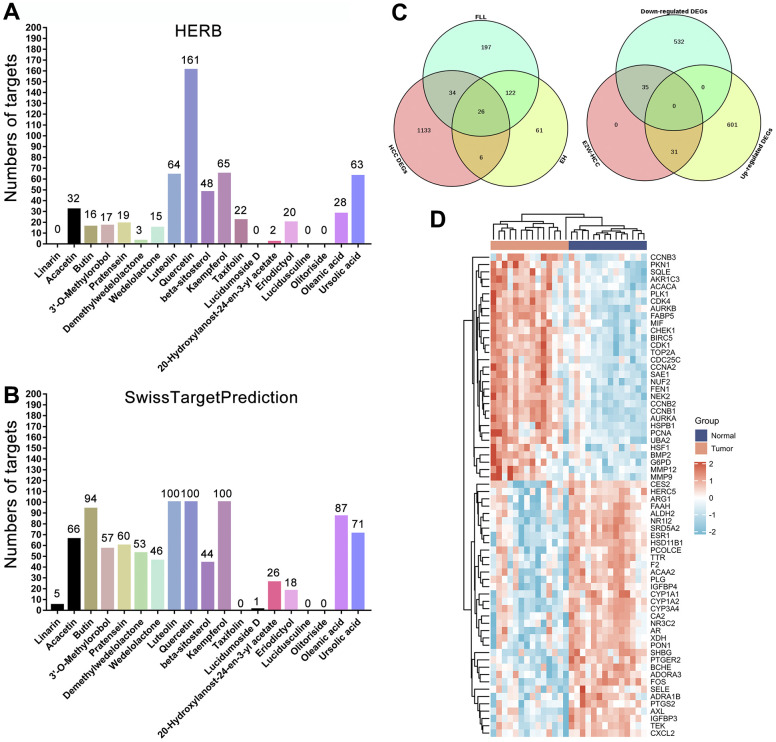
**Prediction and screening of potential targets of EZW for the treatment of HCC.** (**A**, **B**) Prediction and collection of potential targets for EZW based on the HERB and SwissTargetPrediction databases. (**C**) Venn diagram identifying 66 potential therapeutic targets for EZW in the treatment of HCC (35 targets were downregulated and 31 were upregulated in HCC). (**D**) Heatmap analysis of the expression patterns of 66 potential therapeutic targets of EZW in the treatment of HCC in the GSE84402 dataset.

We identified potential targets for these 19 active compounds through two target prediction databases, High throughput Experiment- and Reference-guided (HERB) and SwissTargetPrediction. In the HERB database, flavonoids (luteolin, quercetin, and kaempferol) possessed more targets, but targets failed to be identified for 4 active compounds (linarin, lucidumoside D, lucidusculine, and olitoriside) (Figure 4A and Supplementary File 3). Similarly, in the SwissTargetPrediction database, flavonoids (luteolin, quercetin, and kaempferol) also possessed more targets, while targets for taxifolin, lucidusculine, and olitoriside failed to be identified (Figure 4B and Supplementary File 3). After removing duplicate values, 215 potential targets of 9 active compounds in EH were identified from the two databases. Likewise, 379 potential targets of 12 active compounds in FLL were identified. Finally, 446 potential targets of 19 active ingredients in EZW were identified.

### Target screening, network, and topological analysis of EZW in the treatment of HCC

To explore which pathological targets EZW acts on to treat HCC, we performed Venn diagram analysis of 1199 DEGs in HCC with potential therapeutic targets of the active components in EZW. As shown in Figure 4C, a total of 66 targets overlapped; presumably, EZW may suppress HCC by regulating these 66 genes. Notably, both herbs in EZW act on HCC through 26 common targets, which is speculated to be the reason FLL and EH can exert synergistic anti-HCC pharmacological effects. Moreover, 35 of these 66 genes were abnormally low expressed in HCC and mainly involved metabolism-related genes, including CYP3A4, XDH, ARG1, ADRA1B, and ALDH2. In contrast, there were 31 genes expressed at abnormally high levels, mainly including genes involved in the cell cycle and related to proliferation such as CHEK1, CCNA2, CDK4, CCNB2, CCNB1, CDK1, PCNA, and MMP9 (Figure 4D). EZW may treat HCC by reversing the expression patterns of these genes, but further confirmation is needed.

**Figure 5:**
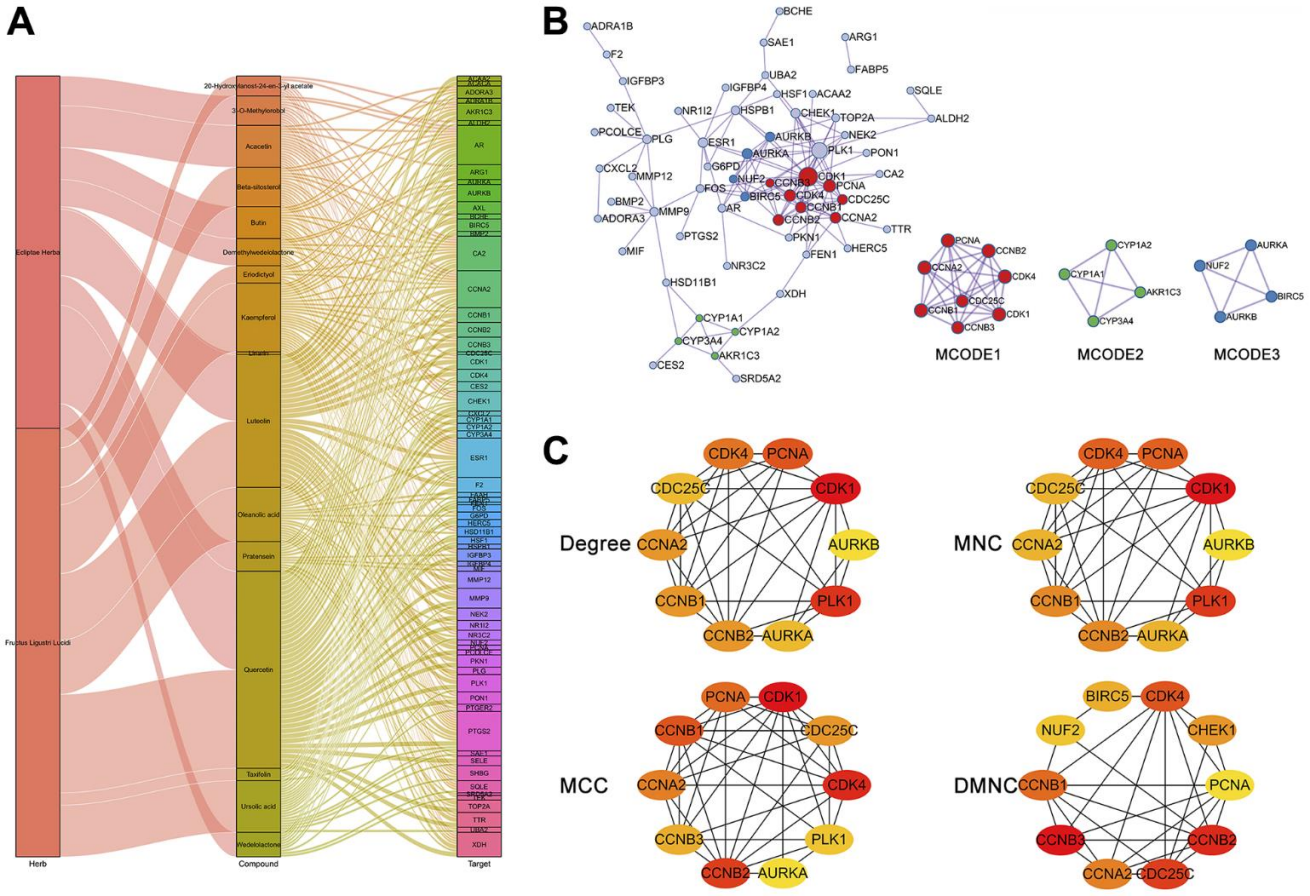
**Network and topological analyses of 66 potential therapeutic targets for EZW in HCC.** (**A**) Herb compound-target network analysis. (**B**) Protein-protein interaction network and gene clustering analysis (Metascape web tool). (**C**) Identification of the top 10 hub genes in the PPI network by different topological calculation methods.

To further understand the interconnections between the herbs, active compounds, and potential therapeutic targets for HCC, we generated an herb-compound-target (H-C-T) network (Figure 5A). The results revealed that the two herbs exerted anti-HCC effects on multiple targets mainly through active ingredients such as quercetin, luteolin, kaempferol, demethylwedelolactone, wedelolactone, oleanic acid, and ursolic acid. Moreover, the results indicated that ESR1, AR, CCNA2, PTGS2, and CA2 were most regulated by multiple active components of EZW. The H-C-T network revealed an intricate molecular network involved in the suppression of HCC by EZW. To further investigate the intrinsic connectivity of the therapeutic targets of EZW against HCC, we generated a protein-protein interaction (PPI) network and performed Minimal Common Oncology Data Elements (MCODE) analysis and annotation on this network. The PPI network contained 60 nodes, 136 connections, and 3 MCODE networks (Figure 5B). We performed pathway and process enrichment analysis for each MCODE component and retained the top 3 terms with the lowest P values as functional descriptions of the corresponding components (Supplementary File 4) MCODE1 was used to annotate cell cycle-related pathways, while MCODE3 was used to annotate metabolism-related pathways.

To explore hub genes in the PPI network, we performed a topological analysis of this network. The PPI network was entered into Cytoscape software to calculate the topological parameters of the nodes in the network. We calculated the top 10 hub genes in the PPI network using a different approach via the CytoHubba plugin and visualized the network via Cytoscape. These calculation methods included the degree method, maximum neighborhood component (MNC), maximal clique centrality (MCC), and density of maximum neighborhood component (DMNC). The results showed that regardless of the calculation method, the genes in the MCODE1 and MCODE2 components of the top ten hub genes were highly enriched, and mainly included CDK1, CDK4, CCNB1, CCNB2, CCNA2, PCNA, AURKA, and AURKB (Figure 5C).

### Functional enrichment analysis of the potential therapeutic targets of EZW for the treatment of HCC

**Figure 6:**
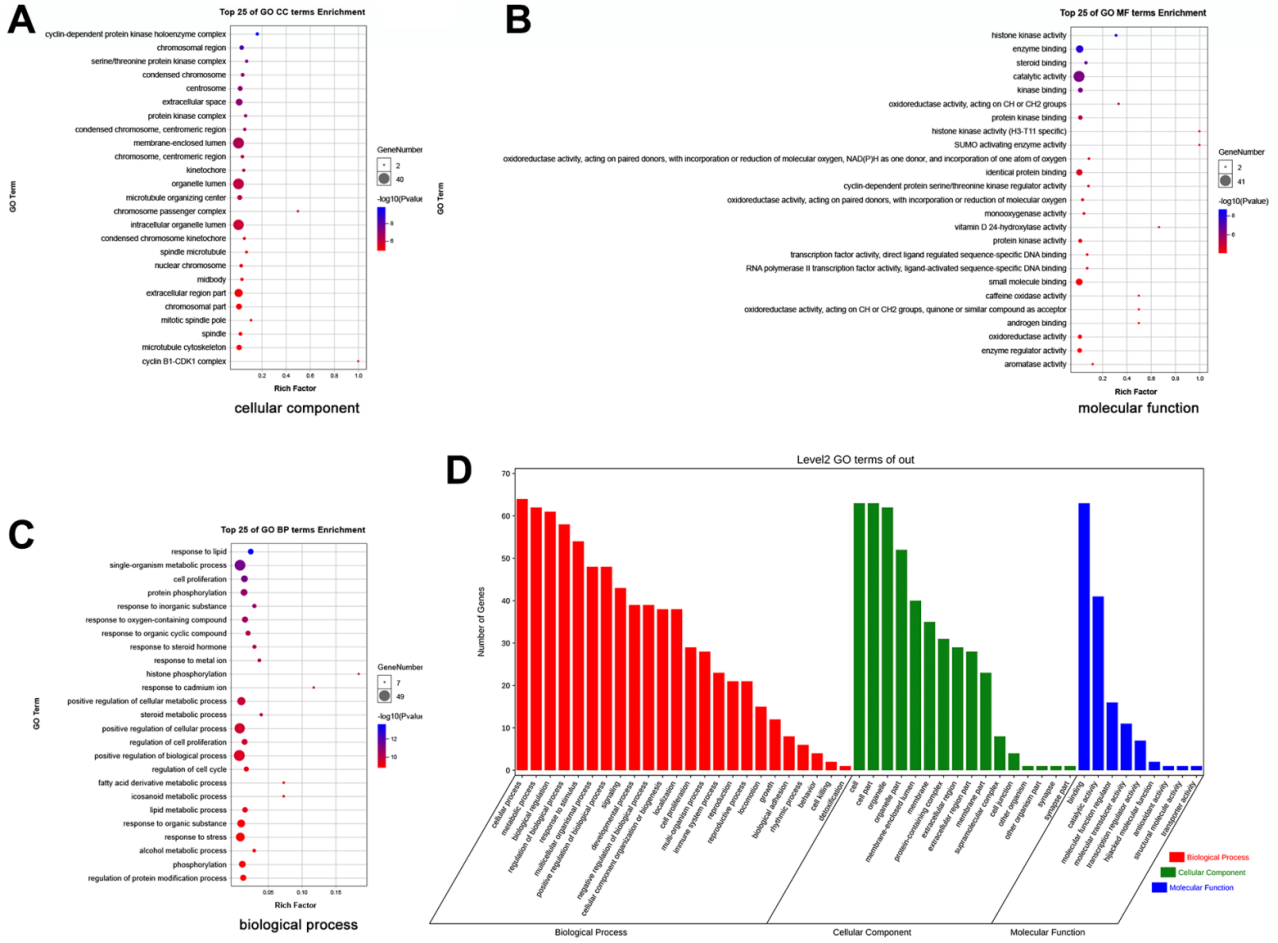
**GO enrichment analysis of 66 potential therapeutic targets for EZW in HCC.** (**A**) Cellular components. (**B**) Molecular functions. (**C**) Biological processes. (**D**) Secondary classification chart of GO enrichment terms.

To fully reveal the mechanism underlying the treatment of HCC by EZW, we performed GO and KEGG pathway analyses. The top 25 GO terms revealed the functions of the potential therapeutic targets of EZW for the treatment of HCC. Annotation analysis of CCs revealed that these therapeutic targets were mainly involved in “cyclin-dependent protein (GO:0000307)”, “serine/threonine protein kinase complex (GO:1902554)”, and “condensed chromosome (GO:0000793)” (Figure 6A). For MF analysis, these therapeutic targets were mainly involved in “histone kinase activity (GO:0035173)”, “enzyme binding (GO:0019899)”, and “catalytic activity (GO:0003824)” (Figure 6B). Moreover, the top 25 GO enrichment analysis results showed that these therapeutic targets were mainly enriched in the following BPs: “response to lipid (GO:0033993)”, “cell proliferation (GO:0008283)”, “G2/M transition of mitotic cell cycle (GO:0044839)”, “regulation of cell cycle (GO:0051726)”, and “fatty acid derivative metabolic process (GO:1901568)” (Figure 6C). Furthermore, we performed a secondary classification of all enriched GO terms, which showed that within the BP category, the GO terms were mainly involved in cellular processes, metabolic processes, and biological regulation (Figure 6D).

**Figure 7:**
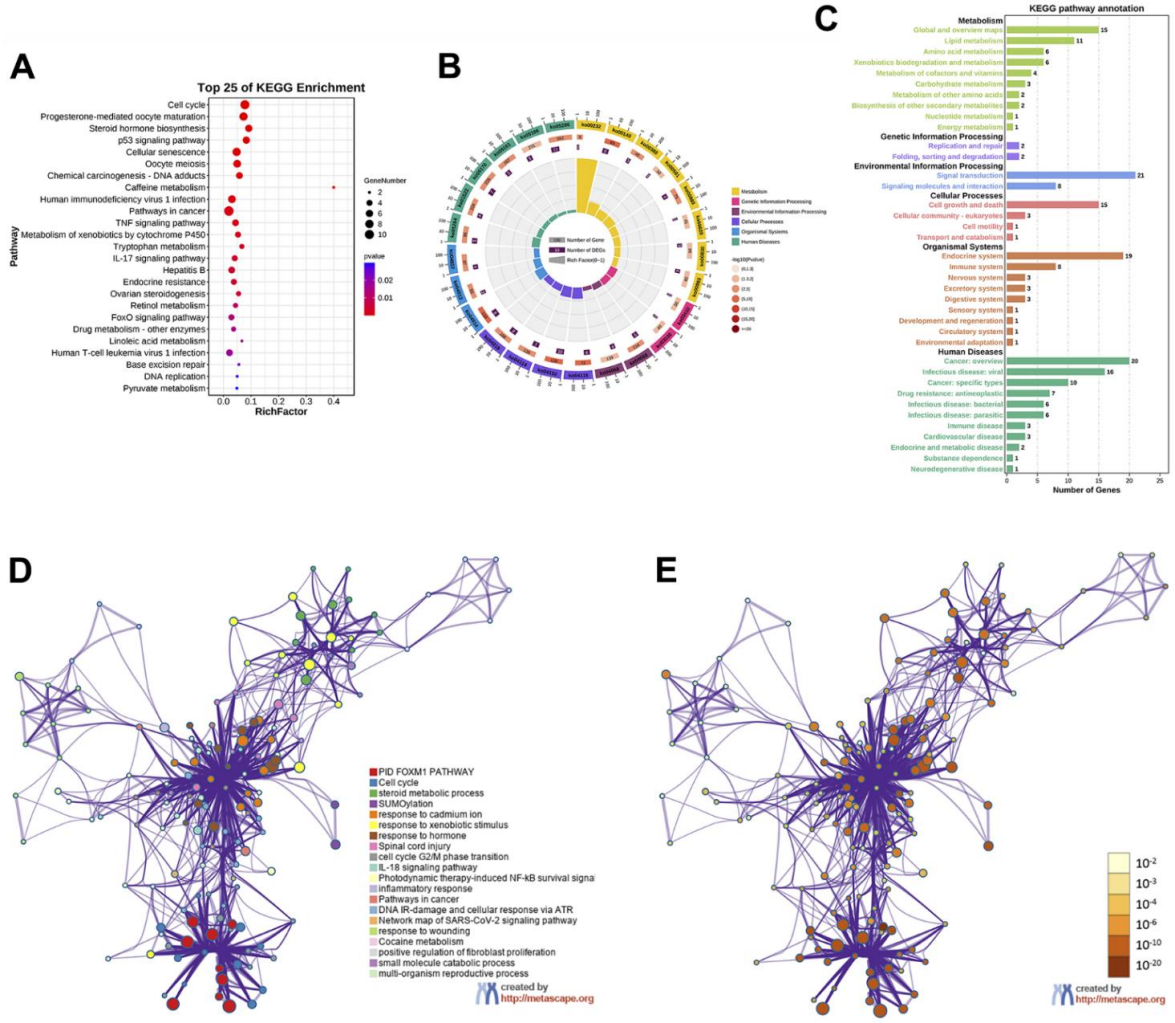
**KEGG enrichment analysis of 66 potential therapeutic targets for EZW in HCC.** (**A**) Top 25 KEGG pathways. (**B**) Secondary classification of the top 25 KEGG pathways. (**C**) Secondary classification of all KEGG pathways. (**D**) Network of enriched terms colored by cluster ID analyzed by the Metascape database, where nodes that share the same cluster ID are typically close to each other. (**E**) Network of enriched terms colored by P value, where terms containing more genes tend to have a more significant P value.

In the KEGG pathway enrichment analysis, the results indicated that EZW exerted its effect on HCC mainly through the following pathways: “Cell cycle (ko04110)”, “Progesterone-mediated oocyte maturation (ko04914)”, “Steroid hormone biosynthesis (ko00140)”, “p53 signaling pathway (ko04115)”, “Cellular senescence (ko04218)”, “TNF signaling pathway (ko04668)”, “Metabolism of xenobiotics by cytochrome P450 (ko00980)”, “IL-17 signaling pathway (ko04657)”, “Hepatitis B (ko05161)”, and “FoxO signaling pathway (ko04068)” (Figure 7A). These top 25 KEGG pathways were mainly divided into 6 categories, including metabolism, genetic information processing, environmental information processing, cellular processes, organismal systems, and human diseases (Figure 7B). To gain a more comprehensive understanding of the mechanism by which EZW suppresses HCC, we next performed a secondary classification of all KEGG pathway annotations, and the results are shown in Figure 7C. In the metabolism category, the KEGG pathways were mainly enriched in “Global and overview maps”, “Lipid metabolism”, and “Amino acid metabolism”. For the cellular process category, the KEGG pathways were mainly enriched in “Cell growth and death”, “Cellular community – eukaryotes”, and “Cell motility”.

To further identify the molecular mechanisms involved in the suppression of HCC by EZW, we captured these therapeutic targets through the Metascape database for pathway and process enrichment analysis. We present the correlations between different enriched terms in a network graph, and the results revealed that “PID FOXM1 pathway”, “Cell cycle”, “steroid metabolic process”, “SUMOylation”, and “cell cycle G2/M phase transition” were significantly enriched (Figure 7D, 7E).

### Identification of cellular senescence-induced/inhibited genes involved in the effects of EZW on HCC and prognostic analysis

Based on the above enrichment analysis, we speculate that cell cycle pathways play a key role in the effects of EZW treatment on HCC. However, recent studies have shown that cellular senescence permanently inhibits the proliferative capacity of cells and induces irreversible cell cycle arrest, which is considered a promising strategy for the treatment of cancer [30]. Our KEGG enrichment analysis results revealed that EZW inhibition of HCC involves the cellular senescence pathway. Thus, we believe that the cellular senescence pathway plays a key role in the suppression of HCC by EZW.

**Figure 8:**
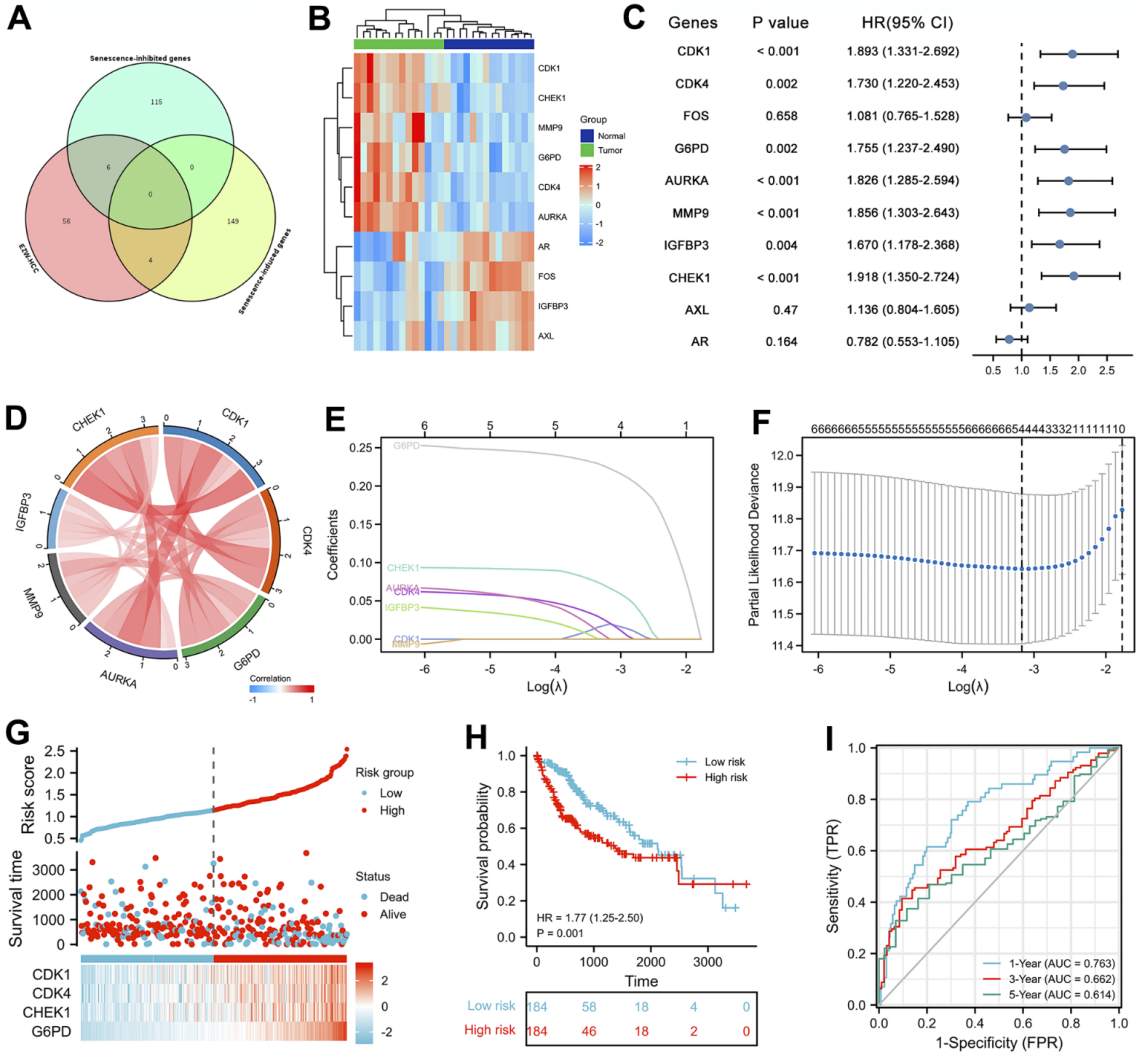
**Prognostic analysis of cellular senescence-related genes and establishment of a prognostic model.** (**A**) Venn diagram identified cellular senescence-related genes among 66 therapeutic targets. (**B**) Heatmap analysis of the expression patterns of 10 cellular senescence-related genes in 14 pairs of adjacent nontumor liver tissues and hepatocellular carcinoma tissues. (**C**) Forest plot of univariate Cox analysis of 10 cellular senescence-related genes. (**D**) Correlation network of 10 cellular senescence-related genes. (**E**) LASSO coefficient profiles of 10 cellular senescence-related genes. (**F**) Cross-validation for tuning parameter selection in LASSO regression. (**G**) The distribution of risk scores, gene expression levels, and survival status of LIHC patients in the training cohort. (**H**) Kaplan–Meier curves of the OS of all LIHC patients in the TCGA cohort based on the risk score. (**I**) Time-dependent ROC curve analysis of the prognostic model (1, 3, and 5 years).

To fully characterize which senescence-related genes are involved as therapeutic targets in the treatment of HCC by EZW, we first identified 153 cellular senescence-induced genes and 121 cellular senescence-inhibited genes from the cellular senescence gene database CellAge (Supplementary File 5). Subsequently, 66 therapeutic targets were assessed by Venn diagram analysis, and 4 cellular senescence-induced genes (IGFBP3, CHEK1, AXL, and AR) and 6 cellular senescence-inhibited genes (CDK4, CDK1, FOS, G6PD, AURKA, and MMP9) were assessed (Figure 8A). Moreover, we comprehensively characterized the expression patterns of these 10 senescence-related genes in HCC (Figure 8B). The results showed that the cellular senescence-induced genes AR, AXL, and IGFBP3 were abnormally expressed at low levels in HCC, while CHEK1 showed the opposite trend. Regarding the cellular senescence-inhibited genes, except FOS, which was abnormally expressed at low levels in HCC samples, they were all abnormally highly expressed.

Univariate Cox proportional hazards regression analysis revealed that seven cellular senescence-related genes were associated with the prognosis of HCC, and CDK1, CDK4, G6PD, AURKA, MMP9, IGFBP3, and CHEK1 were considered risk factors (P<0.01, HR>1) (Figure 8C). Furthermore, the expression levels of these 7 prognostic genes were strongly positively correlated with each other (Figure 8D).

### Establishment of a prognostic risk scores with cellular senescence-related genes in the TCGA dataset

The above seven senescence-related genes were analyzed by least absolute shrinkage and selection operator (LASSO) Cox regression analysis to establish a cellular senescence-related signature for predicting survival. A 4-gene signature was constructed according to the optimum λ value (Figure 8E, 8F). We then established a risk score formula based on the expression of the four genes for patients with LIHC: risk score = (0.0199 + expression value of CDK1) + (0.0210 + expression value of CDK4) + (0.0665 + expression value of CHEK1) + (0.2228 + expression value of G6PD). The risk score of each patient was then calculated using this formula, and patients in the TCGA cohort were stratified into low- and high-risk groups according to the median value of the risk score. The distribution of the cellular senescence-related signature score, the survival status, and a heatmap exhibiting the expression profiles of the 4 genes in the high- and low-risk groups are presented in Figure 8G. Kaplan-Meier survival analysis demonstrated that patients in the high-risk group had a significantly shorter overall survival (OS) times than those in the low-risk group (Figure 8H, HR = 1.77 (1.25-2.50), P = 0.001). Subsequently, time-dependent receiver operating characteristic (ROC) analysis was performed, which showed that the risk score performed well in predicting 1-, 3-, and 5-year OS, with areas under the curve (AUCs) of 0.763, 0.662, and 0.614, respectively (Figure 8I).

### Analysis of the clinical relevance of the 4 cellular senescence-related genes in LIHC

**Figure 9:**
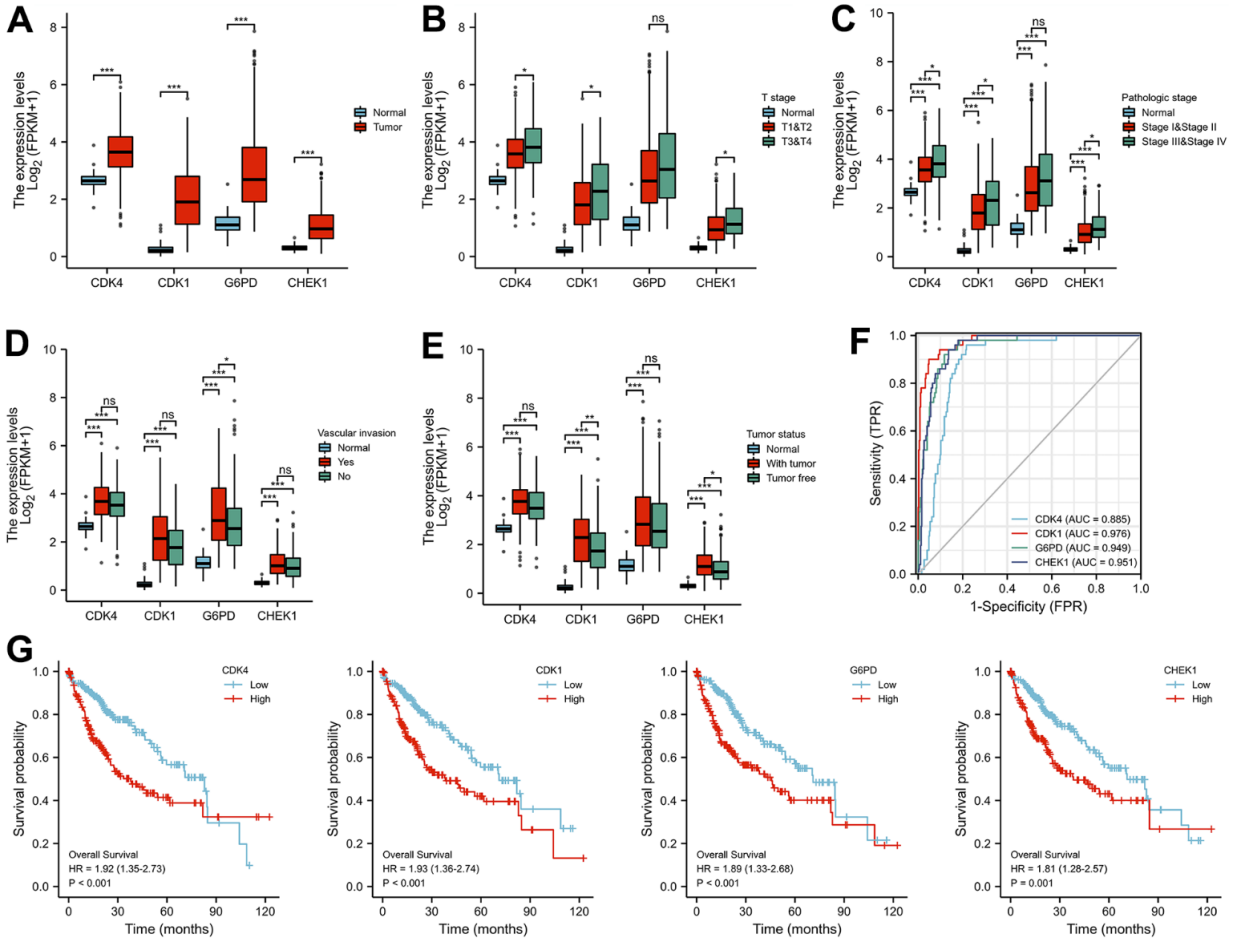
**Correlation analysis of the expression of four key cellular senescence-related genes with the clinical characteristics of LIHC patients.** (**A**) The differential expression of CDK1, CDK4, CHEK1, and G6PD between normal and tumor tissues. (**B**, **C**) CDK1, CDK4, CHEK1, and G6PD mRNA expression in normal individuals or in patients with different T stages (T1&T2 and T3&T4) and pathologic stages (stage I&II and stage III&IV). (**D**, **E**) Differences in the expression of CDK1, CDK4, CHEK1, and G6PD mRNA according to vascular invasion and tumor status. (**F**) Analysis of the AUCs of the 4 cellular senescence-related genes in LIHC. (**G**) Kaplan-Meier curves of OS for different cell cycle-related genes. *, **, and *** represent P < 0.05, P < 0.01, and P < 0.001, respectively.

We analyzed the clinical characteristics of the 4 cellular senescence-related genes (CDK1, CDK4, CHEK1, and G6PD) involved in the treatment of HCC by EZW to further evaluate the clinical application value of EZW in HCC treatment. To investigate the role of these genes in LIHC, we assessed RNA-seq data obtained from 374 HCC patient tissues and 50 normal tissues using transcriptional data from the TCGA-LIHC database. The results showed that the levels of all four genes were significantly higher in tumor tissues than in normal liver tissues (P < 0.05), which was consistent with the previous GEO dataset results (Figure 9A). The transcription levels of the four cellular senescence-related genes in LIHC patients were significantly higher than those in the normal group and were closely related to clinical features such as T stage, pathological stage, and vascular invasion (Figure 9B–9D). Regarding tumor status, the transcript levels of these four genes were downregulated in the ‘tumor-free’ group relative to the ‘with tumor’, while CDK1 and CHEK1 showed significance (Figure 9E). Next, the ROC analysis results showed that the AUCs achieved using the expression levels of CDK1, CDK4, G6PD and CHEK1 were 0.976, 0.885, 0.949 and 0.951, respectively, indicating that these 4 cellular senescence-related genes exhibited adequate predictive performance (Figure 9F). OS analysis showed that patients with high expression levels of CDK4 (P < 0.001, HR = 1.92 (1.35-2.73)), CDK1 (P < 0.001, HR = 1.93 (1.36-2.74)), G6PD (P < 0.001, HR = 1.89 (1.33-2.68)), and CHEK1 (P = 0.001, HR = 1.81 (1.28-2.57)) had poorer OS than patients with low expression levels of these genes (Figure 9G).

### Molecular docking strategy to verify the multicomponent multitarget network of EZW in the treatment of HCC

**Figure 10:**
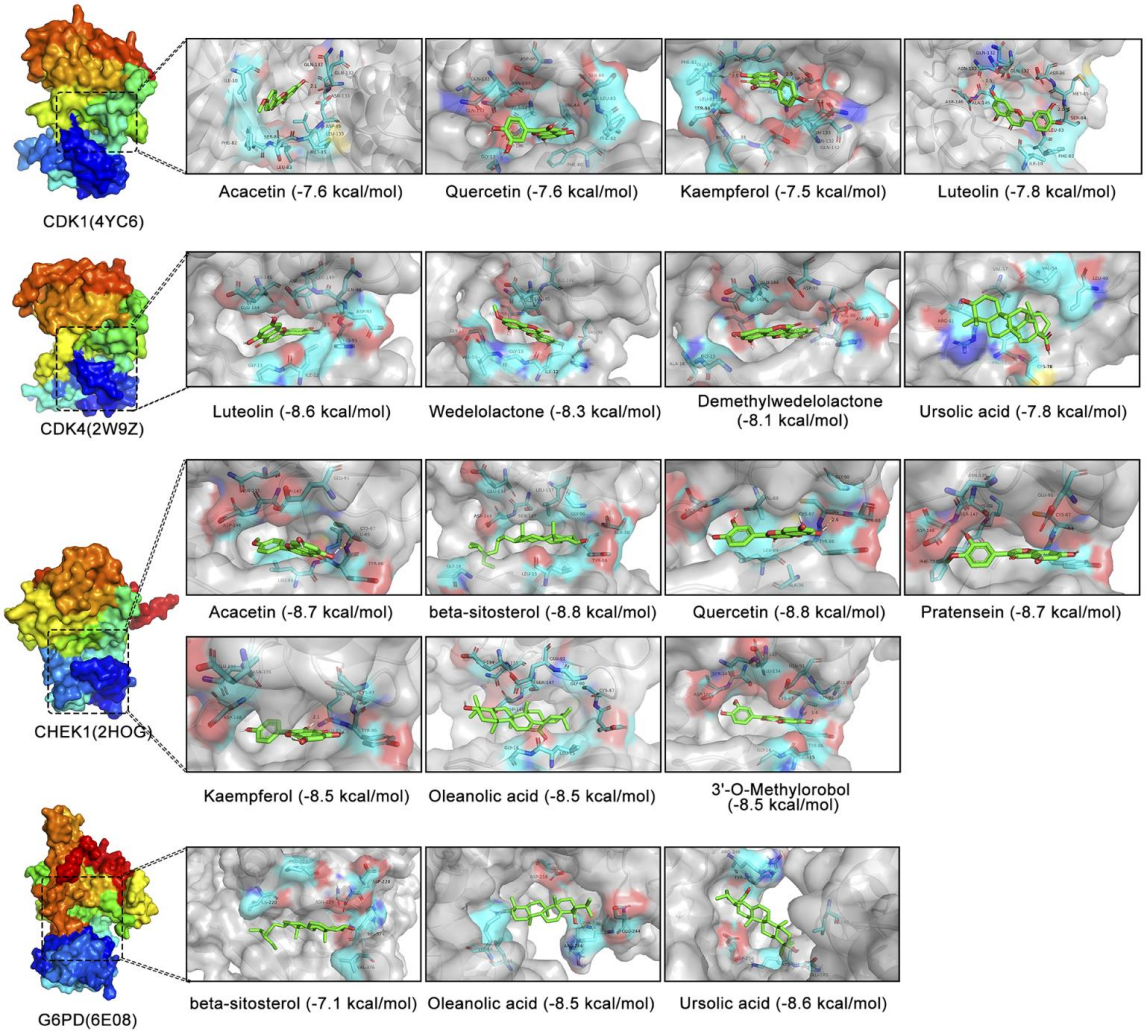
**Molecular docking analysis of different active components in EZW and four cellular senescence-related targets.** Visualization of 3D binding diagrams for protein-ligand predictions based on PyMOL software. Cyan represents the surrounding amino acid residues in the binding pocket, and green represents the active ingredients.

The previous results suggest that CDK1, CDK4, CHEK1, and G6PD are key therapeutic targets through which EZW suppresses HCC. Based on the H-C-T network analysis, EZW inhibits HCC progression through multiple active components acting on these targets. To further reveal how these active ingredients act on these targets, we used a molecular docking strategy to simulate their binding modes and calculated the binding energies to infer the affinity of these active ingredients to the targets. According to the prediction results obtained using molecular docking, a variety of active ingredients (luteolin, quercetin, kaempferol, acacetin, wedelolactone, demethylwedelolactone, ursolic acid, oleanolic acid, pratensein, β-sitosterol, and 3’-O-methylorobol) could bind to these key targets (CDK1, CDK4, G6PD, and CHEK1) with low binding energies (Figure 10). As shown in the binding diagram, these active compounds can bind well in the pockets of these targets and form stable noncovalent interactions with the amino acid residues around the pockets. Presumably, because these active ingredients have multiple hydroxyl groups, they are good hydrogen bond donors or acceptors. Analysis of protein-ligand interactions based on a molecular docking strategy provides evidence for the hypothesis that EZW inhibits HCC by binding to these proteins and inhibiting their activity or expression levels.

## DISCUSSION

HCC is a common malignancy that usually arises in the context of chronic liver disease. Despite modern strategies for patient management, patients with advanced HCC have few treatment options and a poor prognosis. Moreover, the progression of HCC involves multiple mechanisms and aberrant changes in multiple pathological genes [31]. This complexity requires the identification of a therapeutic strategy that modulates multiple targets in HCC to improve patient outcomes. In recent years, the emergence and development of TCM has provided new opportunities for the treatment of HCC. Given that the pathological mechanism of HCC and the multicomponent and multitarget characteristics of EZW are very complex, a single experiment cannot be used to systematically and efficiently reveal the pharmacological mechanism through which EZW suppresses HCC. Through the joint analysis of multiple biological databases, the introduction of molecular docking technology and network pharmacology provides a feasible method for systematically studying the pharmacological mechanism of TCM. Therefore, this study attempted to utilize this comprehensive strategy to explore how EZW exerts pharmacological anti-HCC effects through a multiactive ingredient-multitarget network.

Exploring the pathogenesis of HCC will make an important contribution to providing suitable treatment strategies in the future. In our study, HCC progression was found to be regulated by a complex gene network involving the aberrant expression of thousands of DEGs. Notably, functional enrichment analysis of these DEGs revealed that the pathogenesis of HCC is closely related to various metabolic pathways and processes of cell cycle regulation, which have been shown to be critical for the development of liver disease [32, 33]. Among them, the metabolic pathway (ko01100) in HCC involves abnormal changes in multiple metabolic genes, including high DNMT3A, G6PD, PGP, and EZH2 expression and low AKR1D1, SLC27A5, XDH, LDHD, and ADH3 expression. Chen et al. [34] showed that EZH2 promotes HCC progression by regulating the miR-22/galectin-9 axis. Zhou et al. [35] demonstrated that the long noncoding RNA HCP5 acts as a sponge for miR-29b-3p and promotes liver cancer cell growth and metastasis by upregulating DNMT3A. The study by Cao et al. [36] showed that knockdown of G6PD in HCC reduced tumor volume and tumor weight *in vivo.* Data from Nikolaou et al. [37] suggest that AKR1D1 may play an important role in regulating endogenous glucocorticoid action, which may be particularly relevant to physiological and pathophysiological processes affecting the liver. Chen et al. [38] showed that XDH downregulation promotes TGFβ signaling and the expression of cancer stem cell-related genes in HCC.

Furthermore, cyclins and cyclin-dependent kinases (CDKs) are typically involved in most metabolic processes, such as glucose metabolism, lipogenesis, amino acid metabolism and mitochondrial activity [39, 40]. KEGG pathway analysis revealed that HCC progression was regulated by cell cycle pathways, and multiple cyclins (CCNB1, CCNB2, and CCNB3) and cyclin-dependent kinases (CDK1 and CDK4) were significantly activated in HCC. Previous studies have shown that overexpression of CDKs leads to abnormal cell proliferation and requires CDK activity to respond to DNA damage during DNA replication [41, 42]. Based on these data, designing small-molecule compounds targeting CDKs is considered an effective strategy to treat cancer. Palbociclib, a selective inhibitor of CDK4/6, has been FDA-approved for the treatment of breast cancer and has been shown in multiple studies to be effective in the treatment of HCC [43]. These findings suggest that the key strategy in the treatment of HCC may lie in cell cycle regulation, and the multicomponent-multitarget feature of TCM may be a promising therapy.

The extract of EZW contains a variety of compounds, which undoubtedly increases the difficulty of systematically mapping the pharmacological mechanism of EZW in inhibiting tumors. Therefore, identifying the active components of EZW through databases can be used to more accurately and efficiently elucidate its mechanism. After screening, the key active components of EZW were mainly flavonoids, coumarins, sterols, and natural triterpenoid carboxylic acids. Among them, the flavonoids quercetin and luteolin were identified as the common active components of the two herbs in EZW. We speculate that these two active components may be an important basis for the synergistic anti-HCC effect of the two herbs in EZW. Natural flavonoids have been identified as one of the major classes of natural anticancer agents, exerting antitumor activity through cell cycle arrest as a major mechanism in various cancer cells [44]. Furthermore, multiple active components in EZW have been reported to exhibit substantial anticancer activity in various tumors. Pan et al. [18] showed that coumarin wedelolactone, a characteristic component of EH, inhibited the proliferation of HCC cell lines (HepG2 and Huh-7) by inhibiting the PI3K/AKT signaling pathway. Additionally, demethylwedelolactone, a coumarin component of EH, inhibited the lung metastasis of MDA-MB-231 breast cancer cells in a nude mouse model [45]. Two representative triterpenoids in FLL, oleanolic acid and ursolic acid, were observed to induce apoptosis in various human liver cancer cell lines, indicating that they are potent anticancer agents [46]. These results suggest that the anticancer activities of these active ingredients can serve as an important theoretical basis for EZW in the treatment of liver tumorigenesis.

According to the H-C-T network diagram, EZW may exert its anti-HCC effect through the action of multiple active components on multiple targets. Subsequent PPI network, MCODE, and topological analyses indicated that EZW suppresses HCC through the regulation of multiple cyclins (CCNA1, CCNB1, and CCNB2) and cyclin-dependent kinases (CDK1 and CDK4), which were defined as hub genes. Further KEGG enrichment analysis depicting the top 25 pathways revealed that the cell cycle pathway ranked first, and our aforementioned results indicated that this pathway plays a key role in HCC progression, suggesting that this pathway is an important mechanism by which EZW inhibits HCC. However, the pharmacological mechanism of EZW also involves the p53 signaling pathway, which has been shown to play a central role in regulating the cell division cycle [47]. These findings suggest that EZW may regulate the cell cycle through the p53 pathway, thereby inhibiting HCC. Although these speculations need further verification, they still provide directions for future research on the molecular mechanism of EZW.

Interestingly, the pharmacological mechanism by which EZW inhibits HCC also involves the process of cellular senescence. Cellular senescence constitutes a permanent state of cell cycle arrest in proliferating cells induced by different stresses and has been recognized in recent years as an important mechanism for preventing tumor cell proliferation [30]. TCM with multicomponent and multitarget characteristics is believed to induce cell senescence by activating or inactivating oncogenes, inducing SASP, and triggering DNA damage, thereby inhibiting the occurrence and development of tumors [48]. Leveraging these properties has become a new direction in antitumor research; however, the role of cellular senescence in the treatment of HCC by EZW has been largely underexplored, so a broader understanding of the links among HCC, EZW, and senescence is important. Importantly, we assessed 10 cellular senescence-related genes, including 6 cellular senescence-inhibited genes (CDK4, CDK1, FOS, G6PD, AURKA, and MMP9) and 4 cellular senescence-induced genes (IGFBP3, CHEK1, AXL, and AR). Among them, CDK1, CDK4, G6PD, AURKA, MMP9, IGFBP3, and CHEK1 were considered risk factors in LIHC, and 6 of the 7 genes (CDK1, CHEK1, MMP9, G6PD, CDK4, and AURKA) were abnormally highly expressed in HCC patients. Subsequently, the LASSO Cox regression model and Kaplan-Meier survival curve analysis showed that LIHC patients with high expression of senescence-related genes (CDK1, CDK4, CHEK1, and G6PD) had a poorer prognosis than patients with low expression of these genes. In addition, the expression levels of these genes were closely related to the progression of clinical features such as tumor status, T stage, pathological stage, and vascular invasion of LIHC.

These data strongly suggest that CDK1, CDK4, CHEK1, and G6PD are potential prognostic biomarkers in HCC and key therapeutic targets for EZW to suppress HCC based on the cellular senescence process. Wu et al. [49] reported that blocking CDK1/PDK1/β-Catenin signaling with the CDK1 inhibitor RO3306 could improve the efficacy of sorafenib in the treatment of HCC. A selective CDK4/6 inhibitor, palbociclib, has been shown in recent years to inhibit cell proliferation in human hepatoma cell lines by promoting reversible cell cycle arrest. Moreover, palbociclib alone or in combination with sorafenib, the standard treatment for HCC, impairs tumor growth *in vivo* and significantly improves survival [43]. Previous reports have demonstrated that it is overexpressed and associated with poor prognosis in HCC, suggesting that it is an oncogene and that CHEK1 is negatively regulated by miR-497 and providing a potential molecular target for HCC therapy [50]. Altered metabolism is one of the hallmarks of cancer cells. G6PD levels have been shown to be elevated in many cancers, and it has been shown that G6PD induces epithelial-mesenchymal transition by activating the signal transducer and activator of transcription 3 pathway, thereby promoting HCC migration and hepatoma cell invasion [51]. Although the mechanisms of these genes during cellular senescence remain to be revealed, they still provide potential molecular targets for HCC therapy.

Our study suggests that EZW can be used to treat HCC by acting on these molecular targets, but its intrinsic mechanism needs to be further elucidated. Therefore, *in silico* simulations were used in this study to elucidate the interaction mode between the active components of EZW and these molecular targets. Since the extract of EZW contains various compounds, systematically depicting a clear pharmacological mechanism of how EZW inhibits tumors remains a challenge. The results of molecular docking revealed that multiple active compounds of EZW could be well combined in the pockets of CDK1, CDK4, CHEK1, and G6PD. We speculate that these active compounds may inhibit their activities by targeting these molecular targets, thereby disturbing the cell cycle and metabolism to suppress HCC.

## CONCLUSIONS

Overall, we elucidated the potential pharmacological mechanisms and molecular targets of EZWs in HCC therapy by integrating multiple databases and performing molecular docking analysis. EZW can regulate tumor progression through multiple metabolic pathways, the cell cycle, and cellular senescence. In particular, TCM-induced cellular senescence may become a promising cancer treatment strategy. TCGA data analysis showed that CXP may improve the prognosis and clinical outcomes of HCC patients by regulating cellular senescence-related genes, revealing its clinical application value. In addition, the CDK1, CDK4, CHEK1, and G6PD genes were identified as key therapeutic targets for EZW in the treatment of HCC. Our results suggest that EZW can suppress tumors by disrupting the cell cycle, senescence, and metabolism through these therapeutic targets. To our knowledge, this is the first systematic pharmacological study of EZW in HCC therapy. Therefore, although there are still limitations in this study, this study provides innovative research methods and breakthroughs for TCM research.

## MATERIALS AND METHODS

### Screening the chemical components of EZW

We determined the chemical components of EZW through the Traditional Chinese Medicine Systems Pharmacology Database (TCMSP, https://old.tcmsp-e.com/tcmsp.php). First, the Chinese terms “Mo-Han-Lian” and “ Nü-Zhen-Zi” of *Ecliptae Herba* and *Fructus Ligustri Lucidi* were separately entered into the database to determine their components and retrieve their pharmacokinetic data. Here, we selected two pharmacokinetic parameters as screening criteria to identify the active components in these herbs based on previous studies [26]. Chemical components meeting the criteria of oral bioavailability (OB) ≥ 30% and drug similarity (DL) ≥ 0.18 were considered active ingredients of EZW for subsequent analysis. Moreover, invalid components were removed, including duplicate structures and compounds that could not be retrieved by PubChem (https://pubchem.ncbi.nlm.nih.gov/).

### Screening therapeutic targets of EZW in the treatment of HCC

First, we screened the potential therapeutic targets of EZW’s active ingredients based on the HERB and SwissTargetPrediction databases. HERB (http://herb.ac.cn/) is a high-throughput experiment- and reference-guided database of traditional Chinese medicine. SwissTargetPrediction (http://www.swisstargetprediction.ch/) is an online database that predicts targets of biologically active small molecules. To identify the pathological genes associated with HCC progression, we downloaded GSE84402 from the GEO (http://www.ncbi.nlm.nih.gov/geo/) database. The expression data obtained from 14 pairs of human HCC tissues and corresponding noncancerous tissues were analyzed using the “limma” R package of Bioconductor (https://bioconductor.org/packages/release/bioc/html/limma.html), and the differentially expressed genes of HCC were screened with false discovery rate (FDR) < 0.05 and |log2FC| > 1. Subsequently, the “ggplot2” and “ComplexHeatmap” packages in R language were used for volcano plot and heatmap visualization, respectively. Finally, the potential targets of EZW and the DEGs of HCC were subjected to Venn diagram analysis to obtain overlapping targets. These overlapping targets are the therapeutic targets of EZW in the treatment of HCC.

### Publicly attainable expression datasets

The RNA sequencing (RNA-Seq) expression profile dataset of 374 HCC patients, which included data on clinicopathological characteristics and survival, was downloaded from The Cancer Genome Atlas (TCGA, https://portal.gdc.cancer.gov). Statistical analysis and visualization of gene expression between adjacent nontumor tissues (n = 50) and tumor tissues (n = 374) were performed using the “ggplot2” R package. The list of cellular senescence-related genes was obtained from the cellular senescence gene database CellAge (https://genomics.senescence.info/cells/), including 153 senescence-induced genes and 121 senescence-inhibited genes.

### GO and KEGG pathway enrichment analyses

OmicShare (https://www.omicshare.com/tools), an online data analysis and visualization platform for KEGG pathway and GO enrichment analyses, was used. OmicShare can be also used for Venn diagrams, heatmaps, network building, volcano map analysis, and more. In addition, we used the Metascape (https://metascape.org/gp/index.html#/main/step1) database for pathway and process enrichment analysis and generated network maps.

### Network construction, key module selection, hub gene identification, and topology analysis

A list of 66 therapeutic targets for EZW in the treatment of HCC was entered into the Metascape database (species limited to “Homo sapiens”) to generate a protein-protein interaction (PPI) network. Moreover, the Molecular Complex Detection (MCODE) algorithm was used to identify densely connected network components if the network contained 3 to 500 proteins, as these MCODEs are likely to represent densely connected regions in large PPI networks of molecular complexes. Subsequently, we performed visualization, hub gene analysis, and topological analysis of the raw data (cys format file) of the PPI network obtained by Metascape analysis using Cytoscape software. Through the CytoHubba plugin of Cytoscape software, hub gene analysis was performed on the network, including analysis methods such as degree, MNC, MCC, and DMNC. In addition, the parameters of topological features can be calculated by the Cytoscape plugin Network Analyzer, including “degree”, “intercentrality”, “closeness centrality”, “clustering coefficient”, and “topological coefficient”. The H-C-T network was analyzed and displayed through the OmicShare tool.

### Construction and validation of a prognostic model involving cellular senescence-related genes and LASSO Cox regression

Univariate Cox proportional hazards regression analysis was performed to identify cellular senescence-related prognostic genes (P < 0.01), and gene interactions were visualized by the R package “circlize”. Next, least absolute shrinkage and selection operator (LASSO) Cox regression was conducted with a random seed using the R package “glmnet” to construct the risk score model for predicting survival in the training cohort. Normalized expression matrices of candidate prognostic cellular senescence-related genes were set as independent variables in the regression, and the response variables were OS and patient status in the TCGA cohort. A risk score was determined for each patient based on the normalized expression level of each gene and its corresponding regression coefficient. The formula is as follows:


$$\text{Risk}\;\text{score}=\sum_{\text{i}=\text{1}}^{\text{n}}\text{Coefficient}(\text{i})*\text{expression}\;\text{level}(\text{i})$$

The patients were then divided into low-risk and high-risk groups based on the median risk score.

### Molecular docking analysis

The X-ray crystal structures of the selected proteins were obtained from the Protein Data Bank (PDB, https://www.rcsb.org/), and water molecules and heteroatoms were removed by PyMOL 1.8 software. Moreover, the 3D chemical structures of the active ingredients of EZW were downloaded in SDF format from PubChem and converted to ‘pdb’ format by PyMOL 1.8. Next, the proteins and active ingredients were converted to ‘pdbqt’ format files by AutoDockTools (version 1.5.6), and the grid box feature of AutoDockTools was used to define specific docking pockets in the selected proteins to which the active ingredients could bind. Once all data were prepared, the command prompt was used to perform molecular docking analysis and visualize the docking results with PyMOL.

### Statistical analysis

Data analysis and graph generation were all performed in R version 3.6.3, GraphPad Prism 7.0, and the OmicShare webtool. The statistical significance of normally distributed variables was analyzed by unpaired Student’s t test, and the Wilcoxon rank sum test was used to assess nonnormally distributed variables. ROC curves for 1-, 3-, and 5-year survival to evaluate the predictive efficacy of the risk score were generated using the “timeROC” R package. Moreover, the ROC curves of different genes were analyzed by the “pROC” package and visualized with the “ggplot2” package in R. Kaplan-Meier survival curves for overall survival (OS) analysis were drawn using the R package “survminer”. Two-tailed P values < 0.05 were considered statistically significant.

### Data availability

Publicly available datasets were analyzed in this study. This data can be found here: TCMSP, TCGA, GEO, etc.

## Supplementary Material

Supplementary File 1

Supplementary File 2

Supplementary File 3

Supplementary File 4

Supplementary File 5
