# Supplementary material for: Multilevel data integration and molecular docking approach to systematically elucidate the underlying pharmacological mechanisms of Er-Zhi-Wan against hepatocellular carcinoma
Source: Aging (Albany NY). 2022 Nov 7;14(21):8783–804. doi: 10.18632/aging.204369 (PMC9699749; doi:10.18632/aging.204369)
Supplement: Supplementary File 2 [file aging-14-204369-s002.pdf]

Supplementary File 2. Active compounds of EZW and their parameter information obtained from TCMSP.

Ecliptae Herba											
**Mol ID**	**Molecule name**	**MW**	**AlogP**	**Hdon**	**Hacc**	**OB (%)**	**Caco-2**	**BBB**	**DL**	**FASA-**	**HL**
MOL001790	Linarin	592.6	-0.18	7	14	39.84	-1.68	-2.77	0.71	0.27	16.07
MOL001689	acacetin	284.28	2.59	2	5	34.97	0.67	-0.05	0.24	0.35	17.25
MOL002975	butin	272.27	2.3	3	5	69.94	0.3	-0.4	0.21	0.4	16.8
MOL003389	3'-O-Methylorobol	300.28	2.05	3	6	57.41	0.45	-0.38	0.27	0.32	17.31
MOL003398	Pratensein	299.27	1.37	2	6	39.06	0.39	-0.09	0.28	0.06	17.13
MOL003402	demethylwedelolactone	302.25	1.1	4	7	72.13	0.04	-0.69	0.43	0.34	9.17
MOL003404	wedelolactone	314.26	2.73	3	7	49.6	0.32	-0.45	0.48	0.29	9.61
MOL000006	luteolin	286.25	2.07	4	6	36.16	0.19	-0.84	0.25	0.39	15.94
MOL000098	quercetin	302.25	1.5	5	7	46.43	0.05	-0.77	0.28	0.38	14.4
Fructus Ligustri Lucidi											
**Mol ID**	**Molecule name**	**MW**	**AlogP**	**Hdon**	**Hacc**	**OB (%)**	**Caco-2**	**BBB**	**DL**	**FASA-**	**HL**
MOL000358	beta-sitosterol	414.79	8.08	1	1	36.91	1.32	0.99	0.75	0.23	5.36
MOL000422	kaempferol	286.25	1.77	4	6	41.88	0.26	-0.55	0.24	0	14.74
MOL004576	taxifolin	304.27	1.49	5	7	57.84	-0.23	-0.8	0.27	0.39	14.41
MOL005146	Lucidumoside D	568.63	0.43	4	13	48.87	-1.08	-1.8	0.71	0.23	3.2
MOL005169	(20S)-24-ene-3β,20-diol-3-acetate	486.86	7.34	1	3	40.23	1.09	0.58	0.82	0.23	9.14
MOL005190	eriodictyol	288.27	2.03	4	6	71.79	0.17	-0.54	0.24	0.38	15.81
MOL005209	Lucidusculine	401.6	1.46	2	5	30.11	0.16	-0.39	0.75	0.22	10.55
MOL005211	Olitoriside	696.87	-0.5	7	14	65.45	-2.22	-2.92	0.23	0.26	13.15
MOL000006	luteolin	286.25	2.07	4	6	36.16	0.19	-0.84	0.25	0.39	15.94
MOL000098	quercetin	302.25	1.5	5	7	46.43	0.05	-0.77	0.28	0.38	14.4
MOL000263	oleanic acid	456.78	6.42	2	3	29.02	0.59	0.07	0.76	0.25	57.53
MOL000511	ursolic acid	456.78	6.47	2	3	16.77	0.67	0.07	0.75	0.26	NA
