# Supplementary material for: Multilevel data integration and molecular docking approach to systematically elucidate the underlying pharmacological mechanisms of Er-Zhi-Wan against hepatocellular carcinoma
Source: Aging (Albany NY). 2022 Nov 7;14(21):8783–804. doi: 10.18632/aging.204369 (PMC9699749; doi:10.18632/aging.204369)
Supplement: Supplementary File 4 [file aging-14-204369-s004.pdf]

Supplementary File 4. GO enrichment analysis was applied to each MCODE network to extract "biological meanings" from the network component, where the top three terms with the lowest P values were retained.

Network	Annotation
MyList	M176|PID FOXM1 PATHWAY|-20.8;WP179|Cell cycle|-13.3;GO:1903047|mitotic cell cycle process|-13.2
MyList_MCODE_ALL	GO:1903047|mitotic cell cycle process|-18.1;GO:0000278|mitotic cell cycle|-17.2;M176|PID FOXM1 PATHWAY|-16.3
MyList_SUB1_MCODE_1	WP179|Cell cycle|-19.3;hsa04110|Cell cycle|-19.1;GO:0044772|mitotic cell cycle phase transition|-15.0
MyList_SUB1_MCODE_2	GO:0007052|mitotic spindle organization|-10.1;GO:0007088|regulation of mitotic nuclear division|-9.7;GO:1902850|microtubule cytoskeleton organization involved in mitosis|-9.6
MyList_SUB1_MCODE_3	GO:0019748|secondary metabolic process|-11.2;GO:0042572|retinol metabolic process|-11.1;hsa00140|Steroid hormone biosynthesis|-10.8
